# The complete chloroplast genome of *Corydalis wilsonii* N. E. Brown 1903 and its phylogenetic analysis

**DOI:** 10.1080/23802359.2025.2519224

**Published:** 2025-06-18

**Authors:** Feng Han, Tao Dou, Chuan Luo, Xuhong Song, Pinming Li, Maoxiang Lin

**Affiliations:** ^a^ Chongqing Institute of Medicinal Plant Cultivation, Chongqing, China; ^b^ Chongqing Academy of Chinese Materia Medica, Chongqing, China

**Keywords:** *Corydalis wilsonii*, sect. *Thalictrifoliae*, chloroplast genome, phylogeny

## Abstract

Species within *Corydalis* are valued for medicinal and ornamental uses but taxonomic uncertainties persist due to limited genomic data. Here, we present the complete chloroplast genome of *C. wilsonii*, with a quadripartite structure of 191,388 bp and 140 functional genes. Phylogenetic analysis robustly resolves *C. wilsonii* within a monophyletic clade alongside *C. saxicola*, *C. tomentella*, and *C. fangshanensis*, all nested within sect. *Thalictrifoliae* (bootstrap support = 100%). This study expands the chloroplast genomic resources for *Corydalis* and establishes a taxonomic framework to refine species identification and resolve evolutionary relationships within this ecologically and economically vital genus.

## Introduction

*Corydalis* DC., the most species-rich genus in Papaveraceae, comprises approximately 400–500 annual or perennial herbaceous species distributed across the temperate Northern Hemisphere and the tropical montane regions of East Africa (Lidén et al. 1995). It is noted for its brightly colored inflorescences with horticultural potential and its rich alkaloid content, which contributes to medicinal uses in treating hepatitis, neoplasms, musculoskeletal disorders, and cardiovascular diseases (Zhang et al. 2016). However, taxonomic classification within *Corydalis* remains challenging due to high morphological variability and limited genomic data (Cui et al. 2019; Ren et al. 2021), with fewer than one-quarter of species having sequenced chloroplast genomes (Yu et al. 2021; Kim et al. 2023; Liu et al. 2024).

*C. wilsonii* N. E. Brown 1903 is a glaucous perennial endemic to rocky crevices on forested slopes at 1800–3000 m in northwestern Hubei and Chongqing, China (Zhengyi et al. 2008). Traditionally, whole-plant extracts are used for its anti-inflammatory, analgesic, and diuretic effects to treat oral ulcers, hepatitis, dysentery, and bleeding; its vibrant golden-yellow flowers (often with green-tinged petals) underscore ornamental value. In this study, we report and analyze the chloroplast genome of *C. wilsonii*, and investigated the phylogenetic relationship within the genus.

## Materials and methods

### Materials, DNA extraction, and genome sequencing

**Figure 1.**
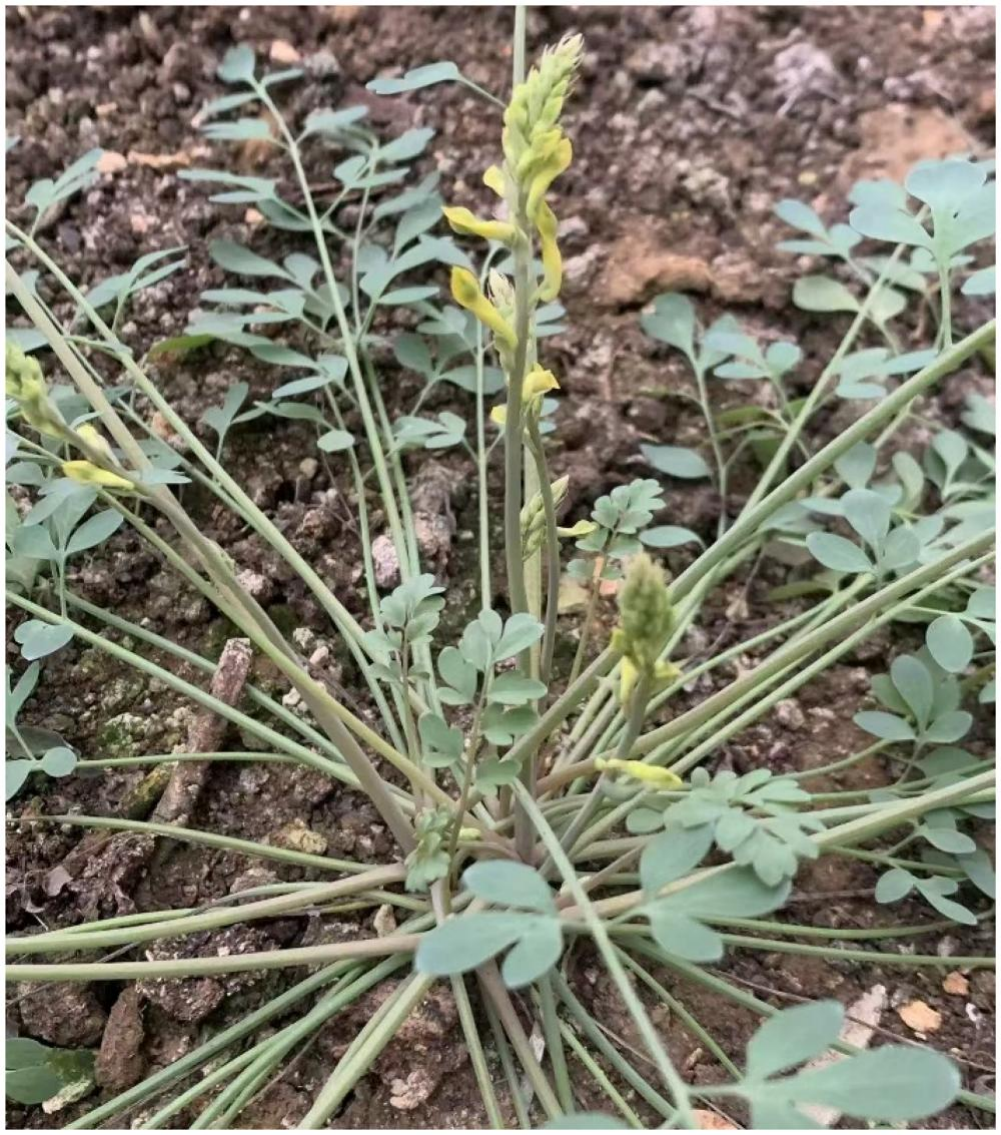
The photograph of *C. wilsonii* was taken by Feng Han without any copyright issues. The plant’s coordinate is 29°8′1.4″N, 107°12′12.34″E. It is a gray-green perennial herb with a height of approximately 15–30 cm, characterized by a taproot and much-branched stems. The leaves are twice-pinnately divided, ovate-lanceolate in shape, with short petioles or nearly sessile. The leaf margins exhibit slight lobing. The racemose inflorescence initially bears multiple flowers densely, which become more spaced as the plant matures. The flowers are golden yellow, with the outer petals displaying green apices. The linear capsule is slightly arcuate, containing glossy, smooth seeds. This species is typically found in rock crevices at elevations of about 3000 m.

The *C. wilsonii* was collected from Nanchuan, Chongqing, China (29°8′1.4″N, 107°12′12.34″E) (Figure 1). A voucher specimen (CQYZS 24031806) was deposited at the Chongqing Institute of Medicinal Plant Cultivation (https://www.cqsywyjs.cn/Index.shtml; contact: Feng Han, hanfengasdf@126.com). Genomic DNA was extracted from 100 mg of leaf tissue using the CTAB method, yielding 419.5 ng/µL DNA (260/280 = 2.07; 260/230 = 1.97). Library construction was performed with the MGI V2 Plus DNA Library Prep Kit (NDM627, ABclonal Biotechnology, Wuhan, China), and sequencing was carried out on the DNBSEQ-T7 platform (Benagen Technology, Wuhan, China). Raw reads were quality-filtered using fastp v0.21.0 (Chen et al. 2018).

### Chloroplast genome assembly and annotation

The chloroplast genome was *de novo* assembled using SPAdes v3.15.4 (Bankevich et al. 2012) with default parameters. Annotation was conducted using GeSeq (Tillich et al. 2017) (https://chlorobox.mpimp-golm.mpg.de/geseq.html) and CPGAVAS2 (http://www.1kmpg.cn/cpgavas2/), predicting protein-coding genes (PCGs), tRNAs, and rRNAs. Manual curation was done using CPStools (Huang et al. 2024) with reference to other Corydalis species. Raw reads were mapped to the assembly using BWA v0.7.17, and coverage depth was calculated with SAMtools v1.9. A circular genome map was generated using OGDRAW (Greiner et al. 2019). Cis(trans)-splicing gene structures and IR boundary junctions were validated with CPGView (Liu et al. 2023) (http://www.1kmpg.cn/cpgview/) and CPJSdraw v1.0.0 (Li et al. 2023).

### Phylogenetic analysis

Sixteen *Corydalis* chloroplast genomes were retrieved from GenBank. Sixty-three orthologous genes were identified via BLASTn (https://blast.ncbi.nlm.nih.gov/Blast.cgi?PAGE=MegaBlast&PROGRAM=blastn&BLAST_PROGRAMS=megaBlast&PAGE_TYPE=BlastSearch&BLAST_SPEC=blast2seq&DATABASE=n/a&QUERY=&SUBJECTS=). Each gene was aligned using MAFFT v7 (Katoh and Standley 2013), and concatenated with PhyloSuite v1.2.3 (Zhang et al. 2020; Xiang et al. 2023). Poorly aligned regions were trimmed with trimAl v1.4.1 (Capella-Gutiérrez et al. 2009). The best-fit model (GTR +F + I + G4) was selected via ModelFinder (Kalyaanamoorthy et al. 2017). A maximum-likelihood (ML) tree was reconstructed using IQ-TREE v1.6.12 with 1000 ultrafast bootstrap replicates, employing *Lamprocapnos spectabilis* and *Fumaria officinalis* as outgroups. Tree visualization was performed in with FigTree v1.4.4 (Rambaut 2018).

## Results

**Figure 2.**
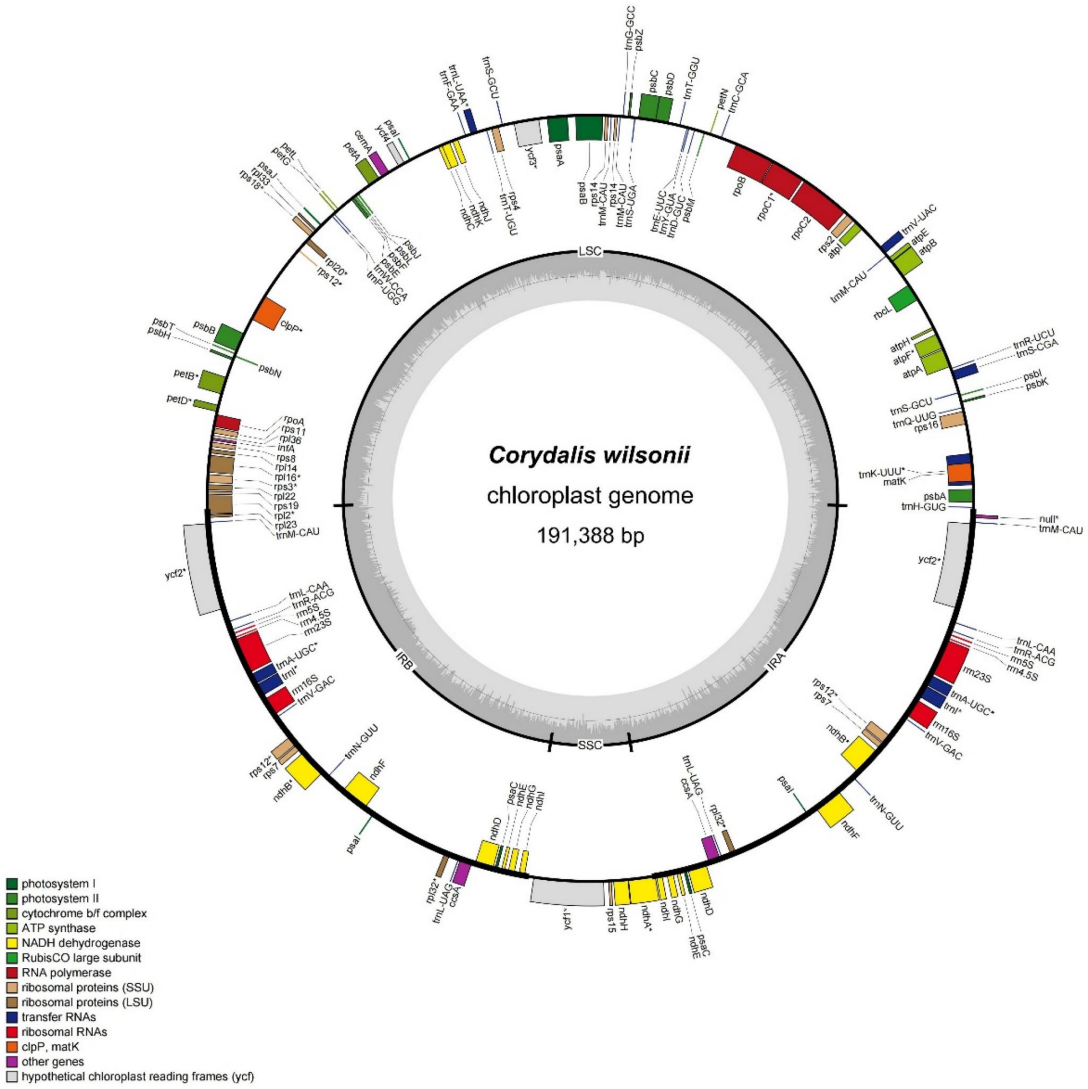
Circular map of the *C. wilsonii* chloroplast genome. Genes with different functions are shown in different colors. Genes on the inner ring are transcribed *clockwise*, while those on the outer ring are transcribed *counterclockwise*. The AT content is depicted as a light gray histogram, and the GC content is represented by a dark gray gradient. Genes containing introns are marked with an asterisk (*).

The chloroplast genome of *C. wilsonii* was successfully assembled and annotated from 17.22 GB of high-throughput sequencing data, with the complete sequence deposited in NCBI under accession number PV339921. This circular genome spans 191,388 bp, characterized by high-coverage sequencing (minimum: ×432, maximum: ×8029, average: ×7918.67), ensuring robust assembly reliability (Figure 2 and Figure S1). Structurally, it exhibits the typical quadripartite architecture: a large single-copy (LSC) region of 97,428 bp, a small single-copy (SSC) region of 9838 bp, and two inverted repeat (IRa/IRb) regions each measuring 42,061 bp. The overall GC content is 40.3%, with regional heterogeneity: IR regions show the highest GC content (42.17%), followed by the LSC (39.13%) and SSC (35.28%).

A total of 140 functional genes were annotated, comprising PCGs, 39 tRNAs, and eight rRNAs (*rrn4.5S*, *rrn5S*, *rrn16S*, and *rrn23S* duplicated). Among the PCGs, 21 cis-splicing genes were identified (Figure S2), including *rps16*, *atpF*, *rpoC1*, *ycf3*, *rps18*, *rpl20*, *clpP*, *petB*, *petD*, *rpl16*, *rps3*, *rpl22*, *rpl2*, duplicated *ycf2* (×2), *ndhB* (×2), *rpl32* (×2), *ycf1*, and *ndhA*. Notably, the *ycf3* gene harbors two introns, while the *clpP* contains three introns. The *rps12* gene exhibits trans-splicing involving two introns. Additionally, eight tRNA genes, namely *trn*K-UUU, *trn*S-CGA, *trn*V-UAC, *trn*L-UAA, *trn*A-UGC (×2), and *trn*I (×2), each possess a single intron.

Comparative analysis of boundary regions among four *Corydalis* species showed that *rpl2* consistently spans the LSC–IRb junction (JLB), with IRb expansion of 149–411 bp. In *C. wilsonii* and *C. fangshanensis*, *ndhF* is located at the SSC–IRa junction (JSA), while in *C. tomentella*, it lies at the SSC–IRb junction (JSB). Gene distribution near IR boundaries shows considerable interspecific variation.

**Figure 3.**
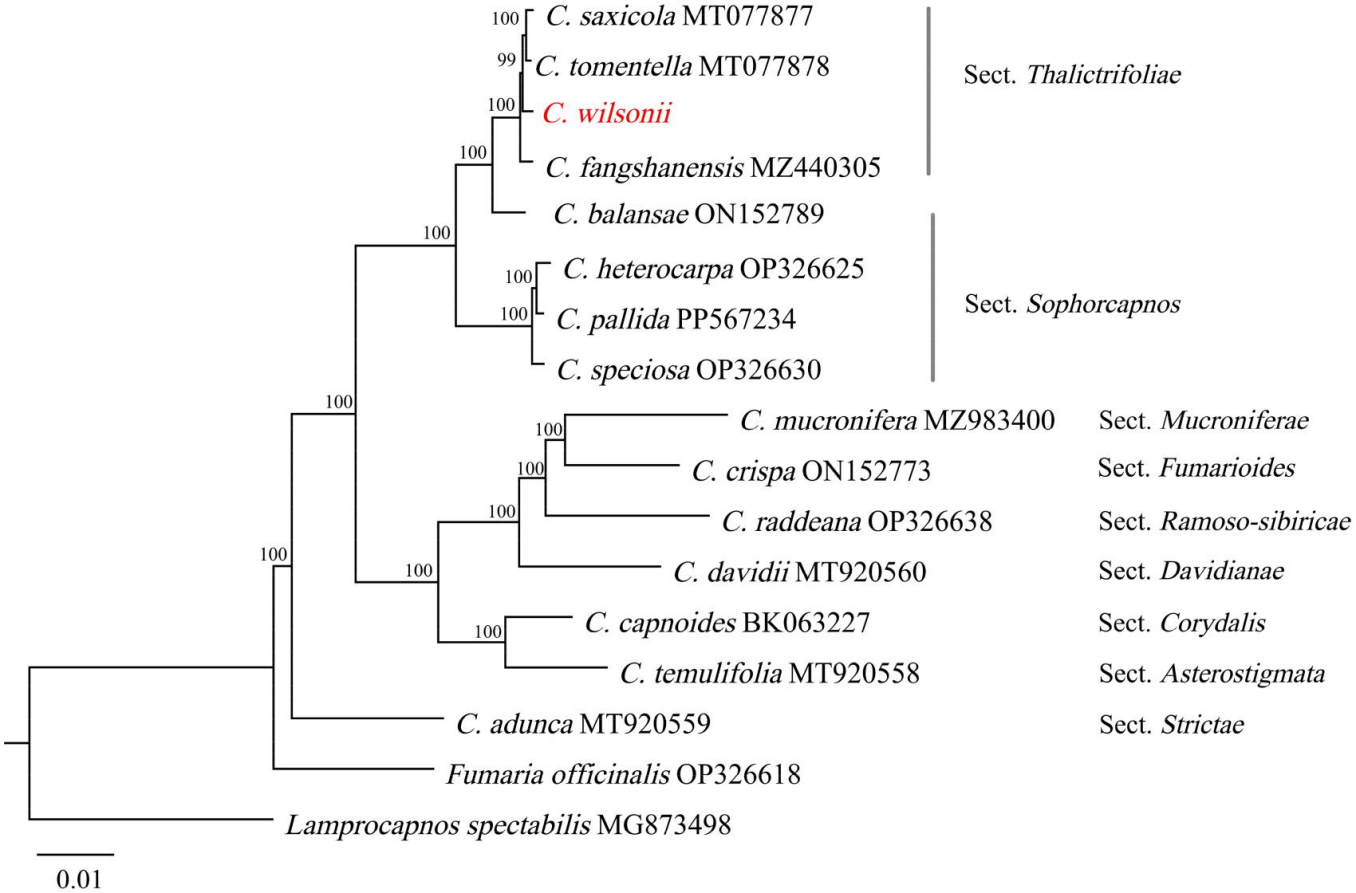
Phylogenetic tree reconstructed using maximum-likelihood (ML) based on 63 orthologous genes from selected *Corydalis* species, with *L. spectabilis* and *F. officinalis* designated as the outgroup. Bootstrap values (≥60%) are displayed above nodes. The specie marked in red is the newly sequenced species in this study (*C. wilsonii*, PV339921). The following sequences were used: *C. saxicola* Bunting MT077877 (Ren et al. 2021), *C. tomentella* Franch. MT077878 (Ren et al. 2021), *C. fangshanensis* W.T. Wang ex S.Y. He MZ440305 (Yu et al. 2021), *C. balansae* Prain ON152789 (Xu et al. 2022), *C. heterocarpa* Siebold & Zucc. OP326625 (Kim et al. 2023), *C. pallida* (Thunb.) Pers. PP567234 (unpublished), *C. speciosa* Maxim. OP326630 (Kim et al. 2023), *C. mucronifera* Maxim. MZ983400 (Raman et al. 2022), *C. crispa* Prain ON152773 (Xu et al. 2022), *C. raddeana* Regel OP326638 (Kim et al. 2023), *C. davidii* Franch. MT920560 (Xu and Wang 2020), *C. capnoides* (L.) Pers. BK063227 (Kim et al. 2023), *C. temulifolia* Franch. MT920558 (Huang et al. 2022), *C. adunca* Maxim. MT920559 (Xu and Wang 2020), *F. officinalis* L. OP326618 (Kim et al. 2023), and *L. spectabilis* (L.) Fukuhara MG873498 (Park et al. 2018).

A concatenated phylogenetic tree (Figure 3) was constructed based on 63 shared chloroplast genes from *C. wilsonii* and 16 additional species. The results demonstrate that *C. wilsonii* clusters with *C. saxicola*, *C. tomentella*, and *C. fangshanensis* within a single clade (bootstrap value = 100%), forming a monophyletic group affiliated with sect. *Thalictrifoliae* Fedde.

## Discussion and conclusions

Similar to other species within the genus *Corydalis*, *C. wilsonii* exhibits significant medicinal and ornamental value, representing an important resource plant. This study investigated the structural characteristics of the chloroplast genome of *C. wilsonii*, revealing a typical angiosperm quadripartite structure with a total length of 191,388 bp encoding 140 genes. Among previously reported *Corydalis* species, *C. edulis* possesses the shortest chloroplast genome (154,395 bp) (Liu et al. 2021), while *C. capnoides* has the largest (199,294 bp) (Kim et al. 2023). *C. temulifolia* contains the highest number of encoded genes (157 genes) among all sequenced *Corydalis* chloroplast genomes (Huang et al. 2022), whereas *C. davidii* and *C. mucronifera* have the fewest (131 genes each). The three sequenced members of sect. *Thalictrifoliae* display chloroplast genome lengths ranging from 189,155 to 192,554 bp, encoding 135–139 genes. *C. wilsonii* conforms to the chloroplast genomic features of sect. *Thalictrifoliae* and represents the member with the highest number of encoded genes within this taxonomic group (Table S1).

Our observations revealed that both the SSC and IR regions of the chloroplast genomes exhibit highly similar sizes among the four sect. *Thalictrifoliae* members, whereas the LSC regions show significant variation (Table S1). Furthermore, distinct differences in IR boundary positions among these members indicate expansion/contraction events of the IR regions into the LSC and SSC regions (Figure S3), which likely contribute to the observed LSC size variation. This phenomenon, widespread in *Corydalis* chloroplast genomes (Xu and Wang 2020), may be associated with their adaptation to diverse ecological niches.

This study constructed a multi-gene concatenated phylogenetic tree based on chloroplast genome data, unequivocally clarifying the taxonomic position of *C. wilsonii* within the genus *Corydalis* at the chloroplast genomic level. In the phylogenetic analysis, the four sect. *Thalictrifoliae* members with sequenced chloroplast genomes formed a robust, well-supported monophyletic clade (bootstrap value = 100%). This topological result is highly consistent with the conclusions of Kim et al. (2023), further validating the feasibility of utilizing chloroplast genomic data to refine *Corydalis* taxonomy and species identification. The publication of this study enriches the chloroplast genomic resources of *Corydalis*, enhances our understanding of evolutionary relationships within the genus and sect. *Thalictrifoliae*, and establishes a critical foundation for future taxonomic and phylogenetic studies in this group.

## Supplementary Material

SM (clean).docx

## Data Availability

The genome sequence data that support the findings of this study are openly available in GenBank of NCBI (https://www.ncbi.nlm.nih.gov/) under the accession number PV339921. The associated BioProject, Bio-Sample, and SRA numbers are PRJNA1238038, SAMN47448210, and SRR32766758, respectively (reviewer link: https://dataview.ncbi.nlm.nih.gov/object/PRJNA1238038?reviewer=iuk646sls4aisi0p793lj1pf8v).
