# Supplementary material for: The complete chloroplast genome of Corydalis wilsonii N. E. Brown 1903 and its phylogenetic analysis
Source: Mitochondrial DNA B Resour. 2025 Jun 18;10(7):610–4. doi: 10.1080/23802359.2025.2519224 (PMC12180353; doi:10.1080/23802359.2025.2519224)
Supplement: SM (clean).docx [file TMDN_A_2519224_SM5760.docx]

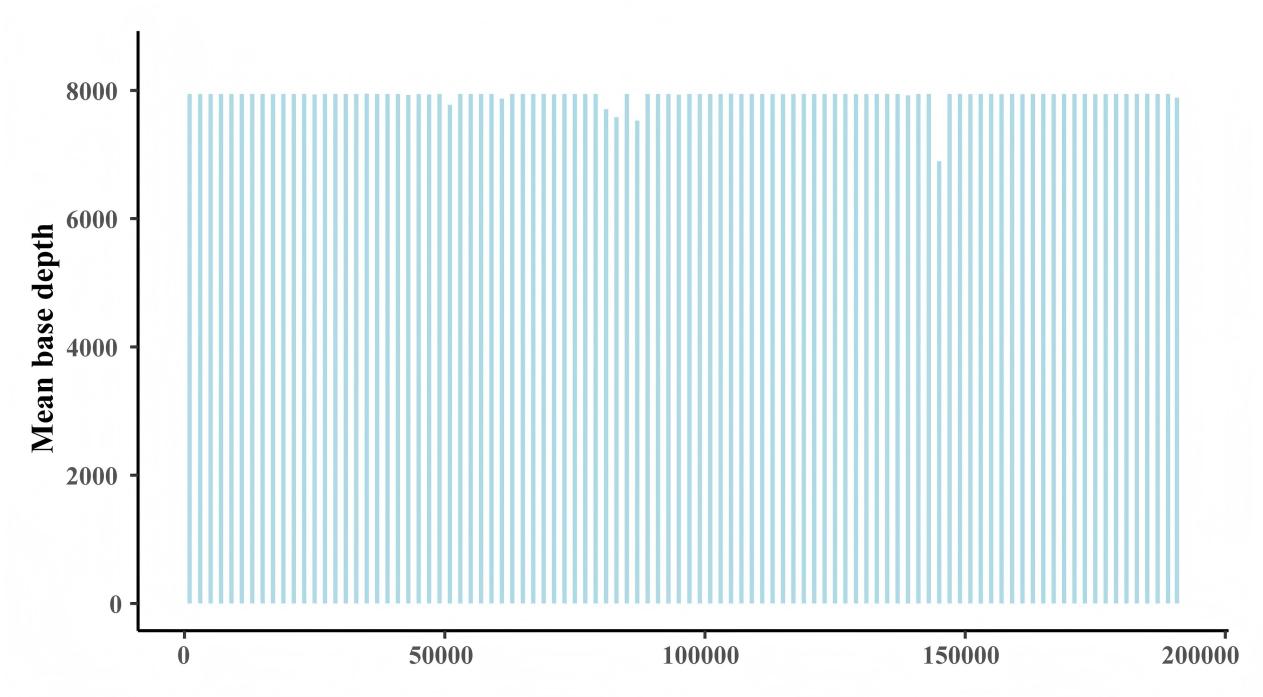


**Figure S1.** The coverage depth map of *Corydalis wilsonii* chloroplast genomes. The height of each bar in the bar chart represents the average coverage depth per 1,000 nucleotide sites. Minimum depth: 432×, maximum depth: 8,029×, average depth: 7,918.67×.


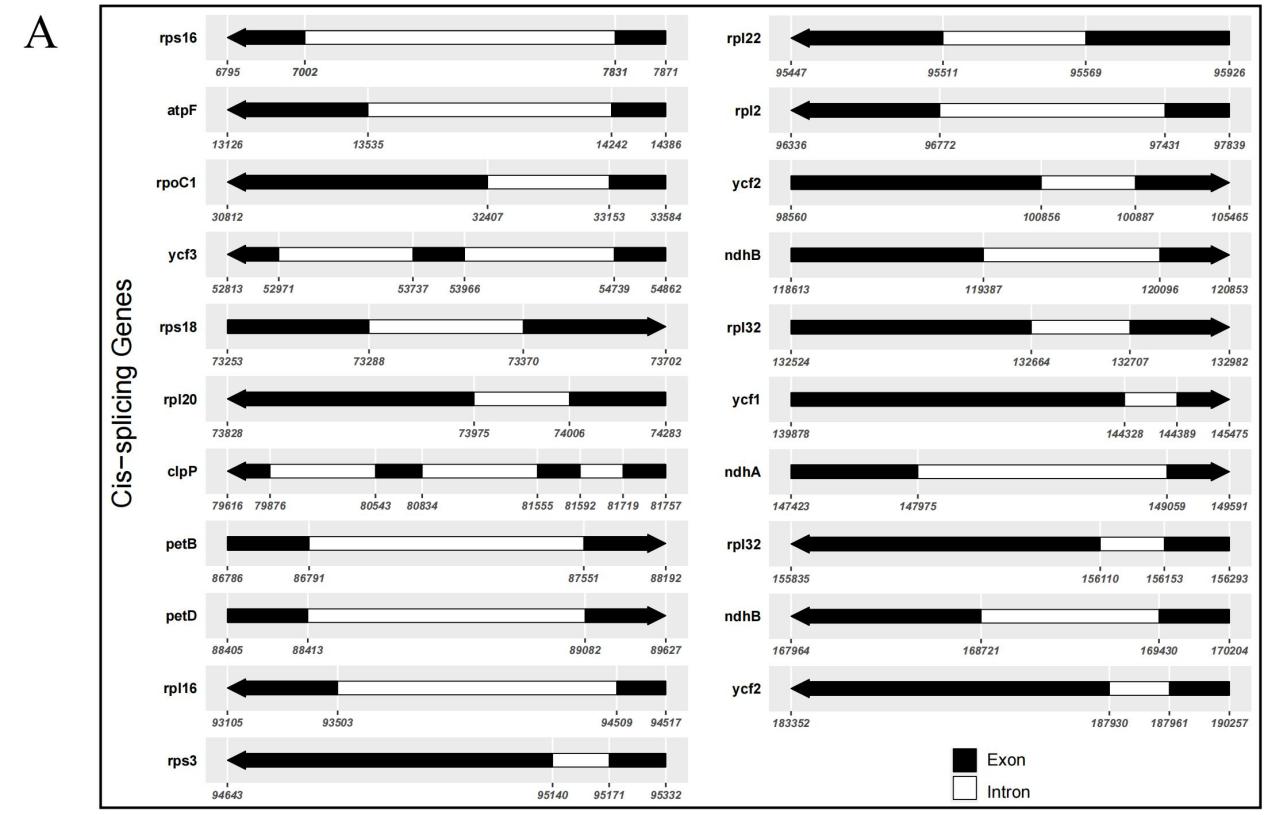


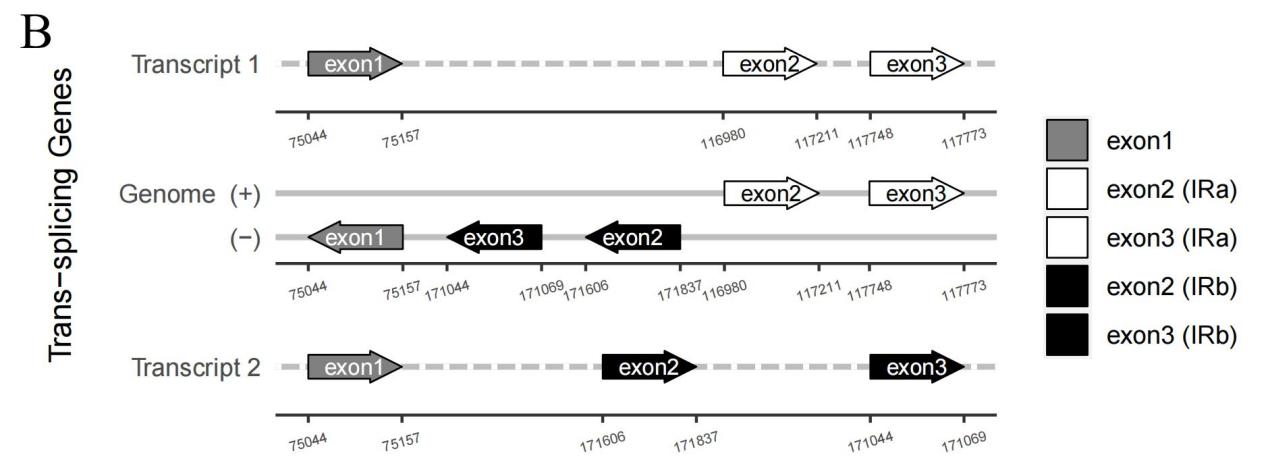


**Figure S2.** The figure displays the cis(trans)-splicing gene structures in *Corydalis wilsonii* chloroplast genome. 21 cis-splicing genes (A) and the trans-splicing gene *rps12* (B) were identified.


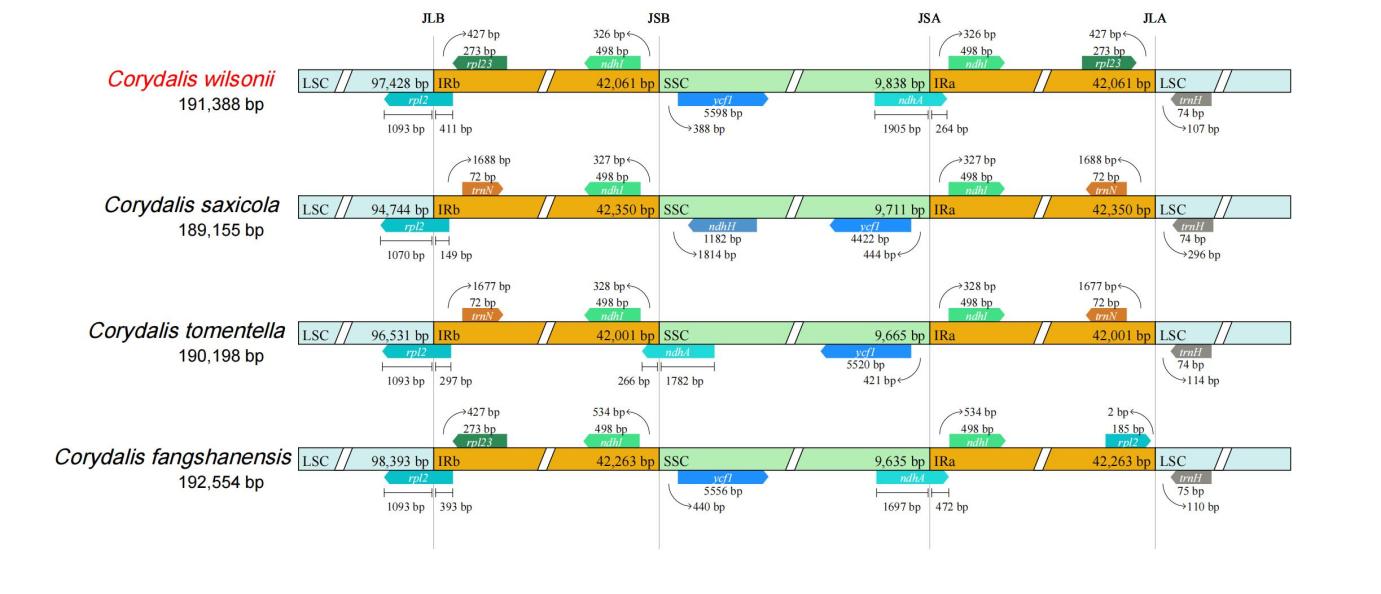


**Figure S3.** Comparison of the IR boundary junctions among chloroplast genomes of *Sect.* *Thalictrifoliae*. JLA and JLB indicate the junction sites between the SSC and the two IRs (IRa and IRb); JSA and JSB denote the junction sites between the SSC and the two IRs.

**Table S1.** Summary of the chloroplast genomes of *Sect. Thalictrifoliae*.

| Species | Length (bp) | | | | Number | | | |
| --- | --- | --- | --- | --- | --- | --- | --- | --- |
|  | genome | LSC | SSC | IR (a, b) | gene | CDS | tRNA | rRNA |
| *C. wilsonii* | 191,388 | 97,428 | 9,838 | 42,061 | 140 | 94 | 39 | 8 |
| *C. saxicola* | 189,155 | 94,744 | 9,711 | 42,350 | 135 | 91 | 36 | 8 |
| *C. tomentella* | 190,198 | 96,531 | 9,665 | 42,001 | 139 | 93 | 38 | 8 |
| *C. fangshanensis* | 192,554 | 98,393 | 9,635 | 42,263 | 138 | 91 | 38 | 8 |
